# The Need for New Biomarkers to Assist with Stroke Prevention and Prediction of Post-Stroke Therapy Based on Plasma-Derived Extracellular Vesicles

**DOI:** 10.3390/biomedicines9091226

**Published:** 2021-09-15

**Authors:** Mircea Popescu Driga, Bogdan Catalin, Denisa Greta Olaru, Agnieszka Slowik, Nikolaus Plesnila, Dirk M. Hermann, Aurel Popa-Wagner

**Affiliations:** 1 Department of Neurology, University Hospital Essen, University of Duisburg-Essen, 45147 Essen, Germany; mirceapopescu100@gmail.com; 2 Experimental Research Center for Normal and Pathological Aging, University of Medicine and Pharmacy of Craiova, 200349 Craiova, Romania; bogdan.catalin@umfcv.ro (B.C.); denissagretaolaru@gmail.com (D.G.O.); 3 Department of Physiology, University of Medicine and Pharmacy of Craiova, 200349 Craiova, Romania; 4 Department of Neurology, Jagiellonian University Medical College, Anny 12, 31-008 Krakow, Poland; slowik@cm-uj.krakow.pl; 5 Institute for Stroke and Dementia Research (ISD), University of Munich (LMU), Feodor-Lynen Str. 17, 81377 Munich, Germany; nikolaus.plesnila@med.uni-muenchen.de

**Keywords:** cerebral ischemia, neurovascular unit, aging, exosomes, biomarkers, therapy, prevention

## Abstract

The risk of having a stroke event doubles each decade after the age of 55. Therefore, it is of great interest to develop neurorestorative therapies of stroke which occurs mostly in elderly people. However, to date, patients at risk for these sequels of stroke are not duly diagnosed and treated due to the lack of reliable biomarkers. Extracellular vesicles (EVs) are lipid bilayer-delimited particles that are shed by the brain cells and are able to cross the blood–brain barrier and enter the blood stream; thus, they may be used to interrogate molecular and cellular events in the brain damaged area. In this review, we summarize the major molecular and cellular responses of astroglia and neurons to cerebral ischemia and assess their impact on post-stroke recovery and rehabilitation. In particular, we ask if EVs secreted by brain cells are responses to cerebral ischemia, and they may shed new light on the interplay between exosomes-mediated interactions between brain cells and the question of how to exploit it in order to predict the individual course of the disease and to introduce specific preventive or therapeutic strategies. Given these findings, we are left with two options: either to (i) transplant neuronal precursors into the damaged cortical area or (ii) to covert abundantly present proliferating astrocytes in the perilesional area into neurons by using recently developed genetic technologies. However, given the complexity of molecular and cellular responses to cerebral ischemia and our limited capabilities to restore brain structure and function, we are left with only one realistic aim: to invest more in prevention.

## 1. Introduction

In Europe, at the beginning of the 21st century, 1,100,000 acute strokes were registered annually, and 80% of them were of the ischemic type [1]. Acute ischemic stroke (AIS) remains as one of the leading causes of disability and mortality in western countries [2]. Cerebral ischemia causes several general and neurological chronic complications, among other physical disabilities in basic activities of daily living (26%), reduced mobility due to hemiparesis (50%), depression (30%), cognitive decline or dementia (50%) and, finally, decreased quality of life in different aspects of daily living [3].

Therapeutic options during the acute phase number in quite a few. Acute occlusions of cerebral vessels can be treated by thrombolysis with rt-PA and/or mechanical thrombectomy; however, very few diagnostic and therapeutic tools are available for stroke survivors who often suffer from long-term disabilities such as sensory-motor dysfunction, reduced mobility, depression, cognitive decline or even dementia, which result in a significantly decreased quality of life.

The risk of having a stroke event doubles each decade after the age of 55 [4]. Therefore, there is of great interest to develop neurorestorative therapies of stroke in older people. Currently, there are a number of pharmacological or cell-based therapies that may improve recovery after stroke. The newly developed therapies, the results of which are currently awaited, were backed by data from experimental studies [5].

### 1.1. The Molecular and Cellular Responses of the Aged Brain to Focal Ischemia

Age is the principal nonmodifiable risk factor for stroke. Pharmacological therapies conducted in models of stroke using young animals have been shown to be efficacious to various extents. However, subsequent clinical studies conducted on older stroke patients failed to improve post-stroke outcomes. This translational failure most likely suggests that using young, healthy animals is not a substitute for old, comorbid animal models of stroke [6,7]. For this and other reasons related mostly to pharmacological approaches and behavioural measures, using old, comorbid animal models has been highly recommended by the Stroke Progress Review Group [8].

Aged animals recover poorly from stroke, and available data indicate that old age is associated with precipitous infarct development that is correlated with increased cellular degeneration, cell death by apoptosis and robust cellular proliferation in the peri-infarcted area. Thus, by using immunohistochemistry we found that most of the BrdU-positive nuclei were identified as activated microglia (45%), oligodendrocyte precursors (17%), reactive astrocytes (23%) and blood cells (CD8+ lymphocytes, 4%). The remaining (<1%) were apoptotic cells [9].

### 1.2. Neurovascular Unit Remodeling in Response to Cerebral Ischemia

The remodeling potential of the NVU serves as an important therapeutic target in stroke and other acute neurologic conditions. Remodeling of ischemic brain tissue involves interactions between neurons, glial and microvascular cells that create a microenvironment that may support tissue and functional recovery. Neuronal outgrowth sprouting of axons and synapses in the damaged area both over long (e.g., along pyramidal tract [10,11]) and short (e.g., within motor cortex [12,13]) distances, thus allowing for the remodeling of neuronal networks that were damaged by the lack of oxygen and nutrients. Remodeling of ischemic brain tissue also includes endothelial progenitor cells (EPC), neural progenitor cells (NPC) and inflammatory cells. New blood vessels are formed, and EPC and NPC are attracted to the stroke lesion. Glial cells not only contribute to necrotic zone confinement by building up of a glial scar but also contribute to the remodeling of the extracellular matrix in the perilesional area. In the process of brain remodeling, the newly formed capillaries play a supportive role by enabling the migration of neural precursor cells via secretion of growth factors [14].

Following stroke, the inflamed endothelial cells secrete pro-inflammatory molecules that leak into the brain parenchyma through the damaged BBB and activate the microglia and astrocytes of the neurovascular unit (NVU) which in turn release pro-inflammatory cytokines (TNFa and IL1b) [15]. Astrocyte activation and proliferations are augmented by the anti-inflammatory cytokine IL-10 secreted by microglia that acts on reactive astrocytes to change the profile of cytokines, represented mainly by TGFb, from a pro-inflammatory to a pro-recovery profile. During the post-stroke brain recovery, the NVU cells release factors (e.g., IL-4, IL-10 and TGFb) that favour structural recovery by switching microglia into a pro-recovery phenotype [15]. Finally, NVU remodeling is paralleled by closing up of the BBB and parenchymal remodeling process, both contributing to restoration of the brain homeostatic environment [13].

#### 1.2.1. Astroglia Responses to Cerebral Ischemia

Aged brains slowly lose neuroplasticity and increased age is a major risk factor for stroke. Although young adult brains may compensate for lost brain cells by recruiting unlesioned neighbouring neuronal networks, unlike young subjects, subcortical strokes in the elderly are often associated with increased demyelination and cognitive decline that cannot be compensated.

Histologically, cerebral ischemia causes the activation of microglia, oligodendroglia and astrocytes. The latter proliferate and migrate rapidly to the lesion site and by day 14 build up a scarring tissue in young animals that is thought to block axonal growth and brain regeneration. Unlike young rats, aged rats show strong and precipitous microglial, astrocytic and oligodendrocytic activation and proliferation that peaked during the first 3 to 7 days after stroke and persisted in aged rats. The early development of the glial scar in aged rats coincided with slow recovery in these animals. Overall, these results suggest that the precipitous, strong gliotic reaction to focal stroke in aged animals is a primary cause of scar tissue buildup that limits post-stroke functional recovery [16].

##### Astrocytic Responses to Cerebral Ischemia in Aged Subjects

Astrocytes are the most abundant cell type in the central nevous system and play an essential role in brain homeostasis by regulating nutrient uptake and BBB permeability. In addition, astrocytes protect neurons from cell death and neurotoxicity by removing waste and play a role in regulating synaptogenesis and neurogenesis [17,18]. Astrocytes have modulate microglial phenotypes and phagocytosis [19]. Astrocytes mediate inflammatory responses by reacting to pro-inflammatory molecules released by microglia in response to brain insults [20].

The number of activated glial cells increases in the normal aging brain. Following cerebral ischemia, astroglia cells rapidly proliferate and migrate into the perilesional area. Simultaneously, neurons are dying in the infarct core and to some extent in the perilesional area, resulting in the disruption of the neurovascular cell balance. This is more evident in the older brains that mount a fulminant inflammatory response to focal stroke in the infarct core and perilesional area [21]. Therefore, restoring the balance between neurons and non-neuronal cells within the post-stroke perilesional area is crucial for post-stroke recovery. The conversion of glial cells to neurons has been pioneered by the group of Magdalena Goetz who succeeded in converting astrocytes into neurons by expressing transcription factors such as Ascl1 or Ngn2 [22]. Further progress has been achieved by overexpressing two transcription factors, Neurog2 and Bcl2 [23]. Quite recently, higher and more reliable adeno-associated viral vectors carrying NeuroD1 transcription factor to convert astrocytes into neurons in the striatum of a mouse model of Huntington’s disease have been described [24]. Adeno-associated viruses (AAVs) are attractive vehicles for transcription factors delivery because they are non-integrating and non-pathogenic. AAVs can transduce both dividing and nondividing cells with sustained expression rates. A second, highly efficient conversion approach using downregulation of a single RNA-binding protein, polypyrimidine tract-binding protein 1 (Ptbp1), using in vivo viral delivery of a recently developed RNA-targeting CRISPR system CasRx; it resulted in higher rates of conversion of astrocytes into neurons [25]. What is noteworthy is that the converted neurons were not only electrophysiologically active, but they also integrated into neural circuitry [23,25].

The second major post-stroke early event is the build up of axonal growth-inhibitory fibrotic scar composed mainly of activated astrocytes surrounded by a large number of activated and phagocytosing microglia cells that may last up to 3 months [26,27]. “Melting” inhibitory astroglial scars has been attempted in animal models for many years without success. However, conversion of proliferating, reactive astrocytes into functional neurons by using retroviral and adenoviral constructs expressing transcription factors is a revolutionary approach for melting the glial scars and circumventing cell transplantation. However, it is not yet established to what extent this new methodology will improve the restoration of structure and recovery of function in the post-stroke fulminant inflammatory environment generated by microglia cells in the brains of aged animal models.

The potential of cell therapy to replace lost neurons is enormous and has direct implications for post-stroke neurorestoration. However, replacing degenerating or dead neurons is not easy because survival and integration of the newly generated neurons in the surrounding neuronal network is dependent on a myriad of factors of which we have little knowledge. Indeed, in preliminary work, we have found that the efficacy of generating new neurons in situ by infecting proliferating astrocytes with a retroviral vector carrying two transcription factors after focal stroke in young and old mice is disappointingly low. We hypothesized that the therapeutic retroviral constructs carrying the transcription factors are engulfed by phagocytic microglia that populate the infarct core and the perilesional area shortly after stroke [28].

##### Microglia Responses to Cerebral Ischemia in Aged Subjects

A severely limiting factor of the genetic conversion of astrocytes into neurons after cerebral ischemia is the phagocytic activity of microglia, which mount an exaggerated inflammatory response to cerebral ischemia, especially in older brains [28]. Moderate insults caused by neuroinflammation may cause the dying neurons to express the “eat-me” signal phosphatidylserine on their membranes. When activated, microglia completely sense phosphatidylserine or the partial phagocytosis (also called “phagoptosis”) of these dying neurons that follows [29]. Neuron engulfment does not discriminate between the neuronal body and therapeutic vector, which will be inactivated too. Therefore, the time to administer a drug therapy should be carefully considered given the phagocytic activity of microglia, especially during the first 7 days of stroke in aged brains [28].

**Figure 1:**
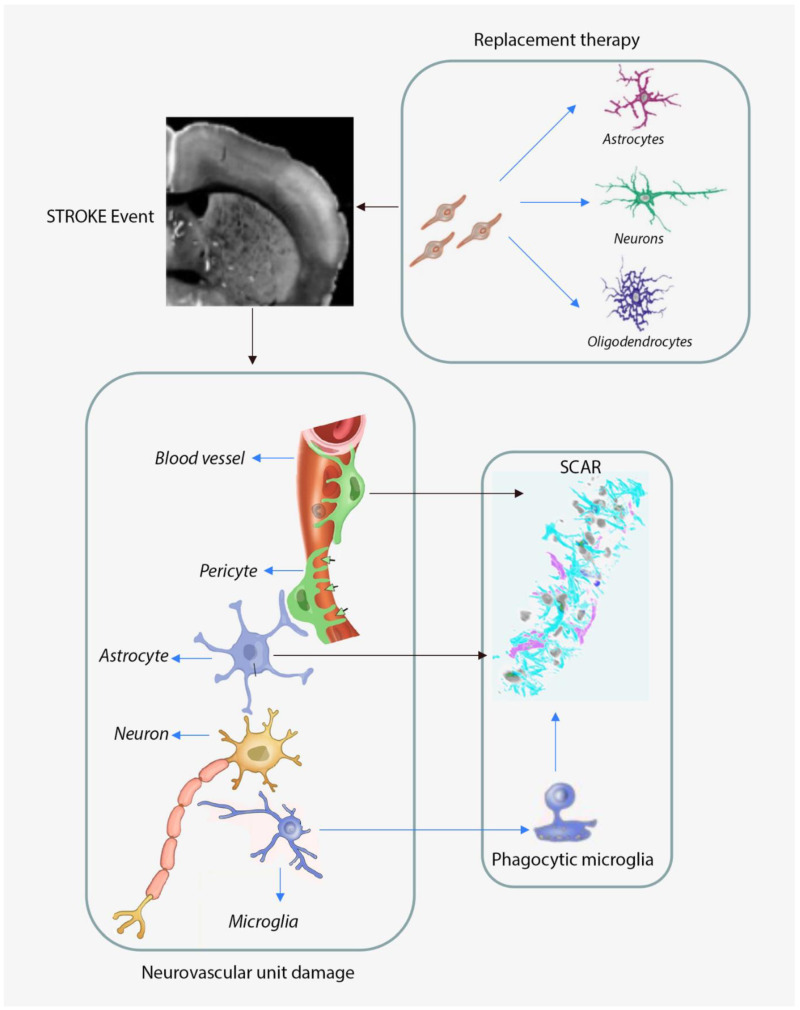
Under severe hypoxic conditions, the neurovascular unit disintegrate, resulting in massive neuronal death. Astrocytes and pericytes will proliferate and rush to the lesion site to build up the glial scar while the microglia will become activated and clear the neuronal and other cellular debris by phagocytosis. Blue arrows refer to cell phenotypic conversion.

As resident immune cells in the central nervous system (CNS), microglia are the first responders to any lesion, including stroke [30]. Within minutes after the onset of an ischemic event, microglia change from their normal surveillance state into a so-called activation state [31]. This allows microglia to migrate towards the lesion, where they start clearing debris, thus helping with tissue repair and remodelling [32,33,34]. However, once at the lesion site, microglia will also start to proliferate and produce pro-inflammatory cytokines, TNFα, IL-1β, IL-1β, IL-23 and IL-12 [35], and cytotoxic substancse including reactive oxygen intermediates, proteinases and complement proteins, resulting in the exacerbation of the tissue injury and appearance of glia scaring, a major obstacle in functional recovery after stroke [36]. As such, the microglia’s role is seen as being both beneficial and detrimental in direct dependance to both the nature and gravity of stroke [37,38]. The NVU responses to a stroke are illustrated in Figure 1.

### 1.3. Current Recanalization Therapies and Rehabilitation Options

Despite intense research performed in the last two decades, for acute stroke patients, there are two treatments options: either major artery recanalization using tissue plasminogen activator to dissolve the thrombus, which can be performed within the first 4 to 6 h after stroke or intra-arterial thrombectomy.

Pharmacological treatment of AIS by intravenous thrombolysis (IVT) and mechanical thrombectomy (MT) of the intracranial artery obstructed by an acute clot are the treatments of choice for AIS. In western countries, reperfusion stroke therapy is applied in up to 30% of stroke victims. Both treatment methods increase the chance for complete recovery and better quality of life for these patients [39]. However, the death rate of the patients treated by reperfusion therapy has remained high at a rate similar to those who are not treated [39].

Intravenous thrombolysis performed by means of plasminogen activators, including tissue type plasminogen activator, promotes fibrinolysis by converting plasminogen into active plasmin, which helps facilitate clot breakdown [40]. This treatment can be delivered up to 4.5 h after stroke onset. Meta-analysis of individual patient data from nine randomized controlled trials showed that the number needed to treat (NNT) in order to achieve good outcomes was 10 for those treated within 3 h after stroke onset and 19 for those treated 3–4.5 h after stroke onset [41]. For patients with unknown time of stroke onset who fulfill the magnetic resonance criteria of the WAKE-UP study (positive diffusion weighted imaging but negative fluid attenuated inversion recovery), this window of time can be prolonged [42].

Intravenous thrombolysis increases the percentage of patients who recover completely after stroke by 30%. It must be considered, however, that the clots responsible for occlusion of the large intracranial arteries are often resistant to intravenous thrombolysis (~70%–80% of cases). What is more is that thrombolysis is associated with low rate of recanalization (<30%), risk of symptomatic parenchymal hemorrhage (~6%) and risk of symptomatic brain edema.

The mechanical removal of the clot from the intracranial artery by means of modern stent retriever devices has been recommended since 2015 for AIS because of occlusion of the proximal large intracranial artery. According to current guidelines, MT should be proceeded by IVT unless contraindications exist [43]. The classic time window for MT is 6 h; however, in patients fulfilling the criteria of the DAWN trial [44] that examined patients presenting between 6 and 24 h after AIS and criteria of the DEFUSE 3 trial that examined patients presenting between 6 and 16 h, the time window for MT can be significantly prolonged [45].

It is estimated that up to 10% of AIS patients fulfill the criteria for undergoing MT [46]. The NNT to obtain functional independence was between 3.2 and 7.4 when compared with best medical treatment [47]. Meta-analysis of individual patient data from the key clinical studies includes the following: MR CLEAN [48], ESCAPE [49], REVASCAT [50] and SWIFT PRIME [51]. EXTEND IA [52] indicates that MT caused a significant increase in the chance for complete recovery (mRS score 0–1; 26.9% versus 12.9%) or independence (mRS score 0–2; 46.0% versus 26.5%) as compared to standardized treatment, including IVT [47]. Despite combined AIS therapy, more than 50% of patients will remain disabled after MT [47].

Rehabilitation after stroke is crucial for improving patient outcomes. However, these procedures are not useful for tissue and functional recovery during the rehabilitation phase. The optimal time to begin rehabilitation after a stroke is still not known. Animal studies have shown that neuroplasticity promotes recovery peaks 7–14 days after a stroke and last for about 1 month [53]. Experts agree that it should be started early and should be focused individually based on patient deficits, including not only physical therapy but also speech therapy, neglect, dysphagia and cognition [54,55].

In clinical practice, physical therapy is mostly used for stimulating post stroke brain recovery probably via the recruitment of neighbouring neuronal circuitries [56,57]. However, physical therapy does not provide a replacement of lost tissue. Therefore, there is an urgent need to develop novel therapies to target the neurorestoration of the damaged brain and functional recovery after stroke. However, the majority of the drugs tested demonstrated safety and therapeutic efficacy in young animal models. However, most the drugs tested in young animals failed to show efficacy when tested in clinical trials. There are two major reasons for the translational failure: aging and associated comorbidities. Indeed, multimorbidity, defined as the presence of two or more chronic conditions, is a norm in older people and highly prevalent in persons with incident stroke, estimated at 89% for those aged ≥65 years and 60% for those aged <65 years. Therefore, it is not surprising that the use of young animals instead of aged, comorbid animals in stroke research largely contributes to translational failure in stroke research [6,7].

### 1.4. Neuronal Replacement Strategies

Neurons are very sensitive to the lack of oxygen supply. In an awake-primate model of cerebral ischemia, 15 to 30 min of interruption in oxygen and nutrients supply will cause neuronal death in the infarct core formerly served by the middle cerebral artery [58]. In the surrounding areas, neurons struggle to survive and can be saved by intra-arterial thrombolysis if performed within 4–6 h after occlusion following CT imaging of the brain.

In rehabilitation settings, physical therapy is used for stimulating post stroke recovery. Indeed, during the first weeks or months after the stroke event, the patients recover motor and cognitive function to some extent by recruitment of neighboring neuronal circuitries, resulting in functional reorganization of the lesioned area. [56,57,59,60,61].

In young animals, spontaneous recovery may occur starting by day 2 after stroke and may reach baseline levels by day 14 depending on the location and size of the ischemic lesion. However, aged rats begin to show delayed improvements of neurological deficits by day 4 or 5 after cerebral ischemia and hardly achieve the baseline levels by day 14 [62].

In animal models, spontaneous recovery occurs if the infarct is in close proximity to the striatum, a subcortical structure that exhibits natural activity-dependent plasticity. It has been shown that this spontaneous recovery could be due to newly born neurons that migrate from the nearby subventricular zone (SVZ). Indeed, it has been shown repeatedly that cerebral ischemia stimulates proliferation of neural progenitors from neurogenic zones such as the SVZ ipsilateral to the lesion in young and older rodents with a maximum at 7–14 days. Proliferating neural progenitors migrate to the peri-infarct striatum along a scaffold of blood vessels over a period of many months [63]. Once it reaches the final location, proliferating neural progenitors may differentiate into spiny neurons and may even integrate into the neuronal network [64].

In humans, functional reorganization has been reported in well-recovered patients with subcortical stroke [57]. Indeed, studies on post-mortem brains provided evidence for enhanced SVZ cell proliferation and neuroblast formation after stroke even in the adult and even in aged humans [65,66]. One study reported an increased number of new cortical neurons originating from the SVZ in the peri-infarct cortex at 65 d after the insult [67]. However, in aged animals, cortical infarcts may not benefit from stroke-stimulated neurogenesis simply because the newly generated neurons in the subventricular zone normally migrate along the rostral pathway towards the bulbus olfactorius and also cannot cross the barrier formed by the corpus callosum [68].

Generation of new cortical neurons has not been unequivocally demonstrated yet in the adult human brain. Thus, a study measuring carbon 14 radioactivity in post-mortem human brain tissue reported that NeuN-expressing cortical cells did not incorporate the C^14^ isotope, which is highly suggestive of discontinued neurogenesis in the human adult brain, at least in cortical areas. A similar study conducted several years later confirmed the lack of neurogenesis after cortical strokes in human subjects [69]. Furthermore, the occurrence of cortical stroke did not appear to induce or increase neurogenesis in humans in a similar analysis conducted several years later. Of clinical interest, in vitro human iPSC-derived cortical progenitors transplanted into human adult cortical tissue survived and gave rise to mature cortical neurons that established synaptic connections with endogenous neurons and exhibited electrophysiological properties similar to those of the host neurons [70]. However, we should be aware that data obtained using animal models may not necessarily translate into a therapy in a clinical setting.

In experimental strokes, ESC-derived or iPSC-derived cortical neurons transplanted into somatosensory injured cortex after an ischemic stroke developed a pattern of connectivity similar to that of endogenous surrounding neurons [71,72]. The transplantation of human-induced pluripotent stem cells improved recovery in cortical stroke survived and differentiated to neurons and ameliorated functional deficits in stroke-injured aged brains [73].

Although preclinical evidence for cell-based therapies is encouraging, it has been estimated that only a few infused cells end up in the ischemic hemisphere after intravenous delivery, where the hostile local hypoxic-ischemic environment causes additional loss of survived cells [74]. Moreover, we, along with others, have shown that only a fraction of transplanted cells will differentiate and integrate into existing neural circuits [73,75]. Since functionality of such transplanted cells has yet to be proven, this technological approach is not ready for the clinic yet.

### 1.5. Prevention Could Be More Successful than Investing in Stroke Therapies

In the last twenty years, considerable efforts have been made to limit the acute neuroinflammation and promote neuronal survival during the recovery phase. However, none of the proposed therapies has been successful translated in clinical trials until now [76,77]. Therefore, our focus has shifted from the acute to the subacute and post-acute phases of stroke in an attempt to improve neurological recovery by stimulating brain plasticity. Indeed, recent studies suggest that, given a permissive microenvironment, extensive remodeling occurs and may take place in the post-stroke adult brain [14,61].

It is well known that the adult brain has a limited capacity to regenerate that is aggravated by ageing, and that intensive rehabilitation is able to improve neurological outcome after stroke; however, providing such treatment for all stroke survivors would place unbearable organizational and financial burden on European health care systems. To overcome this situation, biomarkers need to be developed which, together with the individual risk factors, e.g., age, comorbidities and gender, are able to predict long-term outcome after stroke. Creating such a personalized medicine tool could identify individual patients at risk for bad outcome after stroke and provide them with preventive neurorehabilitation. While improving patient’s lives, such an approach will most likely also reduce health care costs significantly.

Strokes are increasing worldwide in parallel with modernization, changes in lifestyle and the growing elderly population. Major stroke risk factors including ageing and comorbidities; unhealthy diets including high sugar diets; alcohol and tobacco addiction; and obesity have to be overcome by a healthy lifestyle including the Mediterranean diet, healthy dietary components such as polyunsaturated fatty acids and the anti-oxidants curcumin, resveratrol and blueberry polyphenols. However, the best measure to reduce obesity and metabolic inflammation is physical activity. Recently, we summarized the impact of lifestyle on stroke incidence and neurorestauration [78,79,80].

A large amount of money has been invested to compensate for stroke-incurred disabilities and in the development of drugs to enhance neurorehabilitation and nerorestoration after stroke. However, given the complexity of molecular and cellular responses to cerebral ischemia and our limited capabilities to restore brain structure and function, we are left with preventive options including caloric restriction and physical activity, which may counteract ageing and associated neurodegenerative diseases via increased autophagy or increased neurogenesis in the adult brain.

### 1.6. Neurovascular Unit-Derived Exosomes in Response to Stroke

Recent data strongly suggests that exosomes play a critical role in brain homeostasis and plasticity [81] by acting as the bidirectional carrier between neurons, glia, vascular and perivascular cells on one hand and between the brain and periphery on the other [82]. Exosomes are not only involved in epigenetic regulation of communication between neurons and glia cells within the nervous system but also in brain–body epigenetic interconnection mediated by non-coding RNA and miRNA cargo [83].

Astrocytes-derived EVs can selectively target neurons in co-culture, and they can be internalized [84]. Thus, EVs can carry PrP from astrocytes to PrP deficient neurons and thereby act neuroprotectively by enhancing neuronal survival [85]. Likewise, EVs released by astrocytes exposed to oxygen and glucose deprivation (OGD model) can also inhibit neuronal apoptosis and reduce neuronal cell death possibly mediated by miR-92b-3p [86,87]. In vivo, astrocyte-derived EVs have been shown to enhance neuronal survival in animal models of hypoxia, ischemia and hypoglycemia by prion protein (PrP) dependent mechanisms [85].

Pericytes wrap around blood capillaries and use cytoplasmic processes to surround the abluminal surface of the endothelial tube in pre-capillary arterioles, pre-capillary venules and collecting venules. Pericytes are important players in post-stroke NVU remodeling [82]. According to some studies, vascular injury caused by severe hypoxia will activate pericytes which detach from the vascular wall and express nestin, a neuroepithelial stem cell marker capable of conversion into neural and vascular precursor cells [68,88]. Cross-talk between pericytes and endothelial cells occurs through an exchange of exosomes and seems to be is essential for preserving the microvascular structure and function. For example, endothelium or pericyte-derived exosomes were shown to induce angiogenic sprouting in response to hypoxia [89,90]. Evidence indicates that MSCs arise from perivascular cells and pericytes following local injury and perivascular inflammation and secrete exosomes with immunomodulatory and trophic effects, supporting the regenerative microenvironment needed for the post-injury reconstruction of the vascular network, neurogenesis and brain recovery [91].

It is customary to assume that by modulating microglia response to a CNS injury, the neurodegeneration outcome can be changed. Different strategies have been utilised from direct anti-inflammatory therapy [92,93,94,95] to complement modulation [96]. However, the discovery of EVs has allowed for a more dynamic and robust method to modulate microglia activity after stroke, especially as microglia have been shown to rely heavily on EVs to establish long distance communicate with other cells [97]. After it has been demonstrated that stem cells generate their reported effects mainly through EVs [98], a plethora of studies has investigated the impact of neuronal EVs in microglia modulation. Some reports have described that MSC-derived exosomes can attenuate CNS damage by inhibiting the differentiation of activated microglia after stroke [99], while, in an experimental rat model of stroke, EVs containing miR-17-92 clusters and miR-133b have been shown to promote neuronal plasticity and functional recovery [100,101]. Some of the observed effects of microglia-derived EVs are mediated by the C3 complement receptor [102,103]. However, the content of EVs might be different depending on the type of stimuli that microglia are exposed to [104,105]. For example, both BV2 mouse and human C20 cell cultures exposed to EVs obtained from activated and non-activated microglia cells showed an increase in autophagy after being exposed to microglia EVs, regardless if they were obtained from normal or activated microglia [106]. Others have reported a reduction in cellular scaring in mice treated with EVs obtained from non-activated microglia as a regulator of astrocytic activity via the activation of signal transducer and activator of transcription 3 [107].

There is direct evidence that microglia derived EVs not only impact the production of pro-inflammatory mediators (TNFα, IL-1β and miR-155) in traumatic brain injury [108,109], inhibit neuroinflammation and promote neurite outgrowth [110] but also contribute to tau propagation in Alzheimer’s disease [111]. As such, it becomes clear that microglia derived EVS can play a crucial role in future stroke therapies. For example, an ongoing phase two clinical trial is investigating the potential of some MSC derived EVs to reduce stroke sequelae (NCT03384433).

However, microglia activation is far more complex than only a secretory profile [112,113]. As such, our understanding of the complex cellular cross communication between different CNS cells my prove crucial in unlocking our first successful stroke strategy. Neurons-derived exosomes are present both within presynaptic and postsynaptic bodies and are enriched in post-synaptic receptors (e.g., AMPA receptors and GPCRs) [114], thus contributing to neurotransmission and synaptic plasticity within the neuronal network [115]. After a stroke, the activity of glutamatergic synapses is known to be increased by excitotoxic conditions and stimulates neuronal release of exosomes that preferentially bind to neighboring neurons, impacting interneuronal communication [116]. Moreover, the exosomes released secondary to neuronal depolarization seem to promote synaptic plasticity by miRNAs [117].

### 1.7. Brain EVs as Plasma Biomarkers of Cerebral Ischemia

Extracellular vesicles (EVs) are nano-sized particles (40–1000 nm), which are naturally released by cells into the extracellular space. EVs can reach the target cells by fusion with the plasma membrane or by internalization through endocytosis, macropinocytosis or phagocytosis. From there, EVs are able to cross the vessel wall and distribute in various body fluids such as blood and cerebrospinal fluid (CSF). During the process of EV generation, various cellar contents, e.g., mRNAs, miRNAs, proteins and metabolites, are secreted into EVs in a disease-specific manner. Hence, EVs may have the potential to provide information about the physiological status of their parent cells noninvasively [118,119].

**Figure 2:**
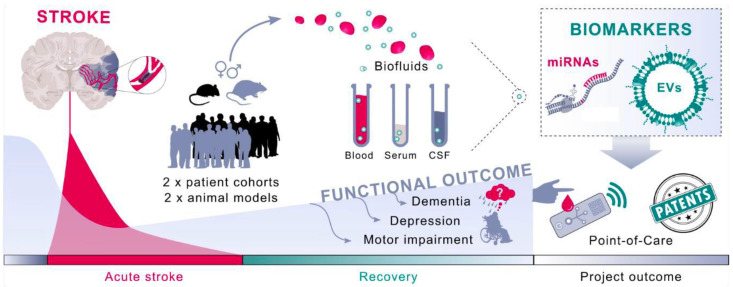
Biofluids (blood and CSF) are sampled from animals and patients acutely after ischemic stroke, and omic profiles isolated are used to develop artificial intelligence-based algorithms able to predict long-term outcomes after stroke in individual patients. Using this approach, we will be able to identify patients at risk for unfavourable outcomes after stroke in the future and direct them towards effective therapies.

EVs could play a critical role in cell signaling events both during the acute phase of stroke and in the long-term post-stroke recovery. The interactions between brain cells are highly orchestrated to perform specific tasks. Neurons, perivascular astrocytes, pericytes and vascular endothelial cells, all part of the NVU, release and uptake exosomes as a mean of intercellular communication during task execution. Of special interest for diagnosis and prediction of post-stroke recovery, brain EVs cross the blood–brain barrier and reach the plasma, allowing assessment of the events occurring in the post-stroke brain [120]. However, given the myriad of post-stroke evens, we can only guess what is really happening with regard to cell–cell interactions and how these interactions can be used to improve stroke [61]; the development of neurorestorative therapies is a true challenge [121]. Indeed, it was demonstrated that brain-derived EVs (Brain-EVs) can be isolated from plasma samples of patient and harness specific disease-related proteins, such as beta-amyloid and phosphorylated forms of tau in Alzheimer’s disease patients [122,123,124]. Thus, disease-specific fingerprint profile of Brain-EVs was isolated from blood and CSF of stroke patients in order to obtain information about the pathophysiological status of the brain after stroke and to predict the outcome of individual patients. This would enable patients at risk for unfavourable outcomes after stroke to be directed to intensive neurorehabilitation, which will eventually improve patient outcomes. The use of new biomarkers to assist with stroke prevention and prediction of post-stroke therapy based on plasma-derived extracellular vesicles is illustrated in Figure 2.

EVs released from the brain cells entering the blood stream have been shown to carry some disease-causing toxic proteins, such as specific miRNA upon ischemic stroke; however, the detailed protein content of exosomes is completely unknown. In recent years, extracellular vesicles (EV) containing microRNAs have been shown to be involved in cell signaling processes triggered by stroke [125,126,127]. However, EVs have never been assessed as potential biomarkers for stroke recovery.

Since the EVs can cross the blood–brain barrier (BBB), obviously these small extracellular vesicles can be detected in the periphery mainly by immune methods, making them extremely useful in deciphering the mechanisms underlying other brain disorders including Alzheimer’s disease, Parkinson’s disease, schizophrenia or bipolar disorders [128,129,130,131].

## 2. Conclusions

Over one thousand drugs have been tested to improve the outcomes of cerebral ischemia. However, not a single one has been shown to be effective. Currently, we believe that one valid therapeutic option is genetic conversion enabling us to manipulate cell fate after cerebral ischemia. However, since the functionality of such transplanted cells has yet to be proven, this technological approach is not ready for the clinic yet. The second valid option, which is also more economical, is to invest in prevention.
